# Global health nephrology education collaboration: a US–Kenya experience

**DOI:** 10.3389/fmed.2025.1647558

**Published:** 2025-10-30

**Authors:** Christopher Owino, Ann Mutugi, Binoy Shah, Mary Gaffney, Mathew Koech, Jie Tang

**Affiliations:** ^1^Moi University School of Medicine, Eldoret, Kenya; ^2^Division of Kidney Diseases and Hypertension, Alpert Medical School of Brown University, Providence, RI, United States

**Keywords:** nephrology education, global health, medical training, international collaboration, Sub-Saharan Africa

## Abstract

**Introduction:**

Chronic kidney disease (CKD) is a growing contributor to morbidity and mortality in Sub-Saharan Africa. In response to regional workforce and training gaps, Moi University (MU) in Kenya and Brown University (BU) in the United States launched a collaborative nephrology education initiative in 2018 to strengthen clinical capacity in Western Kenya.

**Methods:**

In 2019, stakeholder consultations identified key gaps in nephrology education. Targeted interventions were implemented, and a follow-up survey was conducted in 2023 among MU and BU participants to evaluate the program’s impact.

**Results:**

At baseline in 2019, only 9% of 45 surveyed clinicians reported confidence in managing nephrology patients, and 26% had participated in a nephrology education session in the previous 3 years. In response, biweekly virtual case-based conferences were initiated for MU internal medicine residents, who were later invited to join BU’s nephrology conferences and journal clubs. A jointly led annual West Kenya Nephrology Conference began in 2018, and since 2019, a senior BU nephrology faculty member has provided annual on-site bedside teaching. By 2023, 96.6% of respondents reported improved confidence in nephrology care. BU nephrology fellows participating in the collaboration reported enhanced understanding of kidney disease management in global contexts. Planned next steps include community outreach, collaborative clinical and epidemiological research, and the development of the first nephrology fellowship program in Western Kenya.

**Conclusion:**

This collaboration demonstrates a sustainable model for international nephrology education partnerships, with measurable benefits for both institutions. The approach may serve as a blueprint for other programs seeking to build global capacity in nephrology care and training.

## Introduction

Chronic kidney disease (CKD) represents a growing global public health concern, with an estimated worldwide prevalence of 10% (1). Despite a general decline in mortality associated with end-stage kidney disease (ESKD), mortality attributable to CKD overall remains high, as documented by global disease burden studies (2). This burden is disproportionately borne by low- and middle-income countries (LMICs), where non-communicable diseases such as diabetes and hypertension—key drivers of CKD—continue to rise, compounded by a persistent burden of infectious diseases. In Africa, the estimated prevalence of CKD is approximately 14%, significantly higher than the global average (1). In Kenya, the true prevalence remains unclear due to the absence of comprehensive, high-quality epidemiological studies (3).

Globally, the demand for nephrology services is increasing, yet the growth of the nephrology workforce has not kept pace. This is particularly pronounced in LMICs such as Kenya, where the nephrologist-to-population ratio is only 0.7 per million, compared to a global median of 11.8. A key barrier to meeting this demand is limited training capacity. Kenya only established its first clinical nephrology fellowship in 2016 through the East African Kidney Institute in Nairobi (4, 5). Western Kenya, with a catchment population of approximately 25 million people from western Kenya, southern South Sudan, and eastern Uganda, remains without a formal nephrology training program. As of 2018, Moi Teaching and Referral Hospital (MTRH)—the second-largest public hospital in Kenya with 1,300 beds and an academic affiliate of Moi University—had only one nephrologist on staff, responsible for overseeing both inpatient and outpatient general nephrology and dialysis services. This solo nephrologist was assisted by medical officers with only 1 year of postgraduate training. Given the significant shortage of trained nephrology personnel, strategic partnerships are essential to enhance nephrology training in this underserved region.

Since 2018, Brown University has collaborated with Moi University under the Academic Model Providing Access to Healthcare (AMPATH), a consortium that includes MTRH, Moi University, and multiple North American universities. This manuscript describes the initiation, development, and outcomes of a unique nephrology education collaboration between Brown University and Moi University. We present preliminary findings from a needs assessment and follow-up survey of internal medicine residents, the primary target of the educational initiative. We also outline future directions to expand bidirectional educational opportunities and enhance kidney care.

## Materials and methods

**FIGURE 1:**
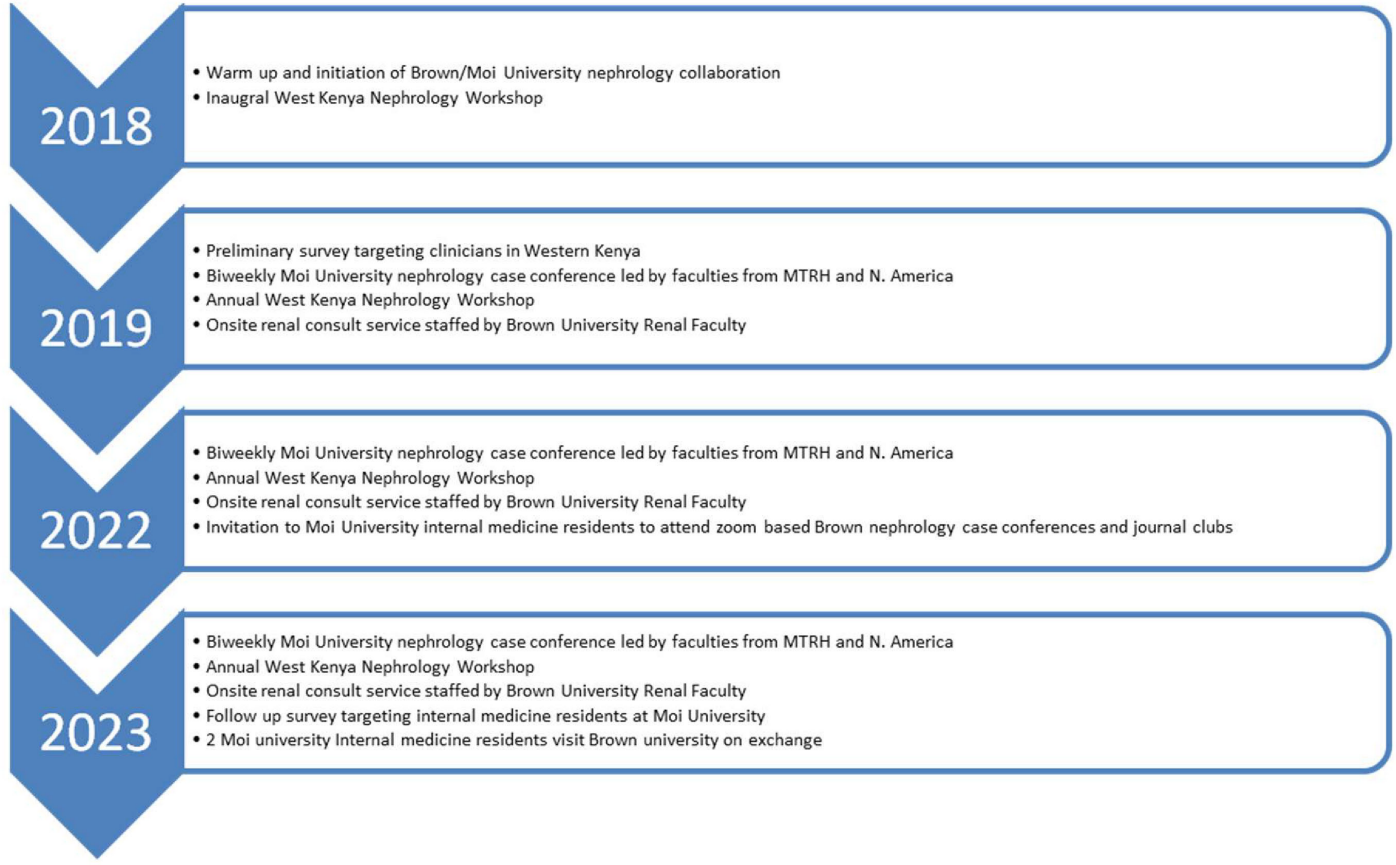
Depiction of key milestones in the Brown/Moi University nephrology education collaboration between 2018 and 2023.

We describe the collaboration between Moi University and Brown University in three phases: initial groundwork, Phase 1, and future directions (Figure 1). We detail the planning and implementation processes and present findings from two needs assessment surveys conducted in 2019 and 2023. These surveys targeted medical trainees in Western Kenya involved in the care of patients with kidney diseases, with no exclusion criteria applied. A separate evaluation surveyed Brown nephrology fellows participating in this program as part of their global health training. All the surveys conducted were anonymous and voluntary.

In 2019, a census survey (Table 1) was conducted among 45 participants at the inaugural West Kenya CME Workshop. The survey included eight items: six binary (yes/no) questions assessing involvement in patient care, exposure to nephrology education, familiarity with Kidney Disease Outcomes Quality Initiative (KDOQI) or Kidney Disease: Improving Global Outcomes (KDIGO) guidelines, comfort in managing CKD patients, and the need for renal physiology education. Two open-ended questions addressed the preferred format of nephrology education and self-reported level of nephrology training, categorized as “little or no training,” “some training but not competent,” or “well-trained.”

**TABLE 1:** Moi University (MU) and US trainee and pre- and post-survey questions.

MU trainee pre- and post-survey questions	US trainee pre- and post-survey questions
Do you feel comfortable taking care of patients with kidney problems in general?	I can describe the differences in disease burden related to CKD) between high, middle, or low income regions and the global challenges in CKD management
Do you feel comfortable taking care of patients with AKI?	I have familiarity to models or systems of CKD care in middle or low-income regions
Do you feel comfortable taking care of patients with electrolyte disorders, i.e., dysnatremia, dyskalemia etc?	I am able to evaluate potential causes (micro and macro) of marginalization and inequity related to kidney care delivery in middle or low-income regions
Do you feel comfortable taking care of patients with acid/base disorders?	I can describe major stakeholders in policy-making and culture barriers of CKD care in middle or low-income regions
Do you feel comfortable taking care of patients on dialysis?	I am able to plan, implement and evaluate an evidence-based program for effective resource utilization and quality improvement in middle or low-income regions
Do you feel comfortable taking care of patients after they received kidney transplant?	
Do you feel comfortable taking care of patients with glomerular diseases?	
Do you have any training on medical genetics?	
Are you familiar with the updated nephrology guidelines i.e., KDIGO?	

In April 2023, a follow-up census survey (Table 1) was conducted among 30 Moi University internal medicine residents who had participated in the Brown-Moi nephrology educational activities, including biweekly case conferences and annual workshops. This survey assessed confidence in managing nephrology disorders, prior training in renal pathology and genetics, access to nephrology literature, mentorship needs, and future educational suggestions. A 10-point Likert scale (1 = low, 10 = high) assessed confidence and training adequacy. Scores ≤ 5 were categorized as “low” and >5 as “satisfactory.” Responses were summarized as frequencies and percentages.

The Brown nephrology fellows’ survey (Table 1) consisted of five items assessing perceived ability to address key global health nephrology concepts in LMICs. These included understanding disparities in CKD burden, care models, policy stakeholders, cultural barriers, and implementation of evidence-based programs. A Likert scale from 0 (no capability) to 5 (full capability) was used, yielding a total possible score of 25. Surveys were administered before and after the Kenya elective, and results were presented as percentages.

## Results

### Preliminary survey (2019)

Of the 45 respondents, 6.7% were medical students, 57.8% recent medical graduates, 35.5% internal medicine trainees. All participants were involved in patient care, including kidney disease management. However, only 26% had received formal nephrology lectures in the preceding 3 years, and just 4% were aware of KDOQI or KDIGO guidelines. Overall, only 4% of the respondents scored above 75% of the maximum score, 90% of respondents scored below 25%.

Regarding nephrology training, in separate interviews, 67% reported little or no exposure, 29% reported some but inadequate training, and only 4% felt well-trained. Only 9% felt comfortable managing kidney disease. Among internal medicine residents (*n* = 16), 83% preferred case-based discussions, and all endorsed the need for sessions on renal physiology.

### Follow-up survey (2023)

**FIGURE 2:**
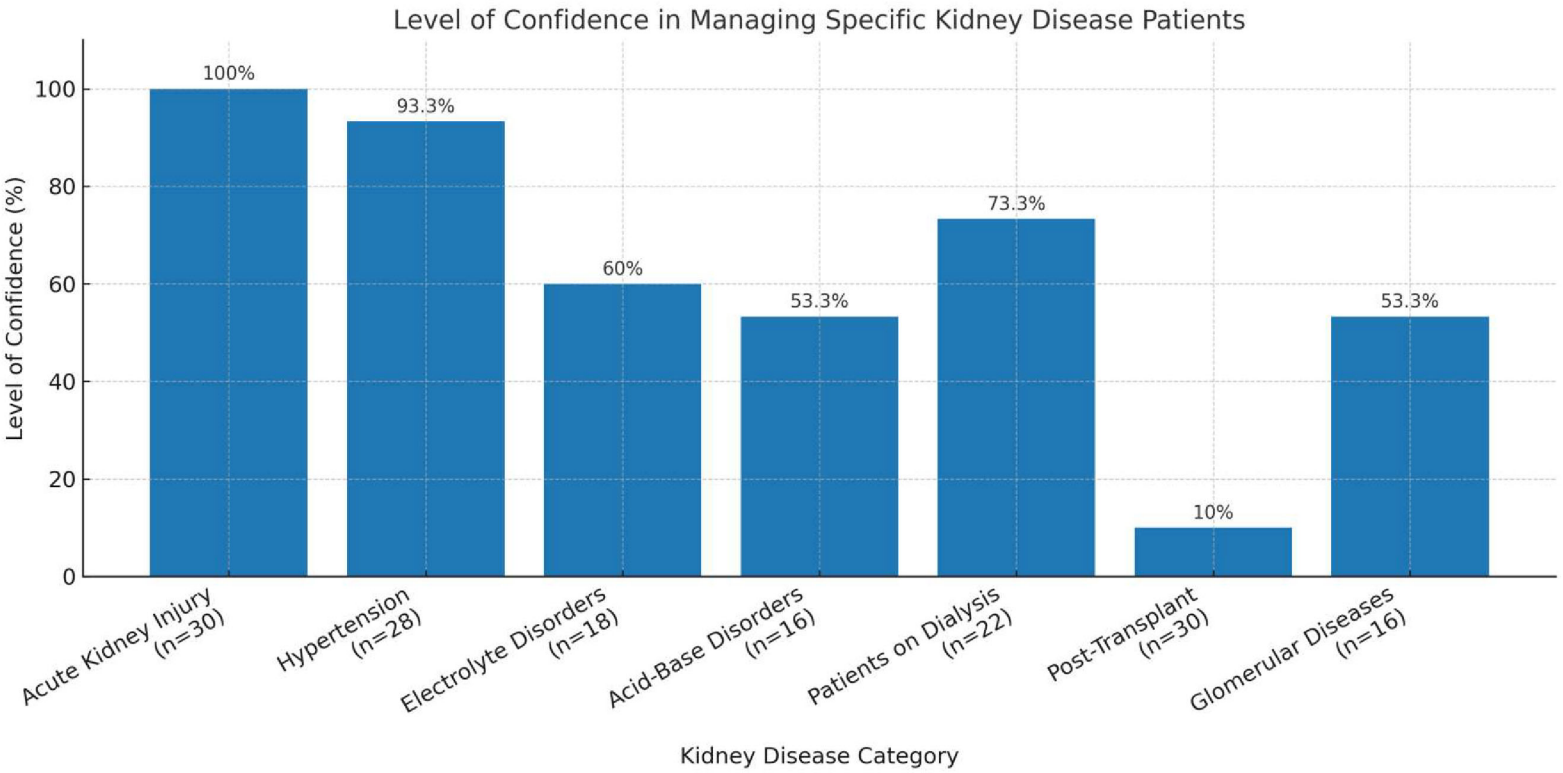
Level of confidence in managing specific kidney disease patients among Moi University internal medicine residents in 2023.

**FIGURE 3:**
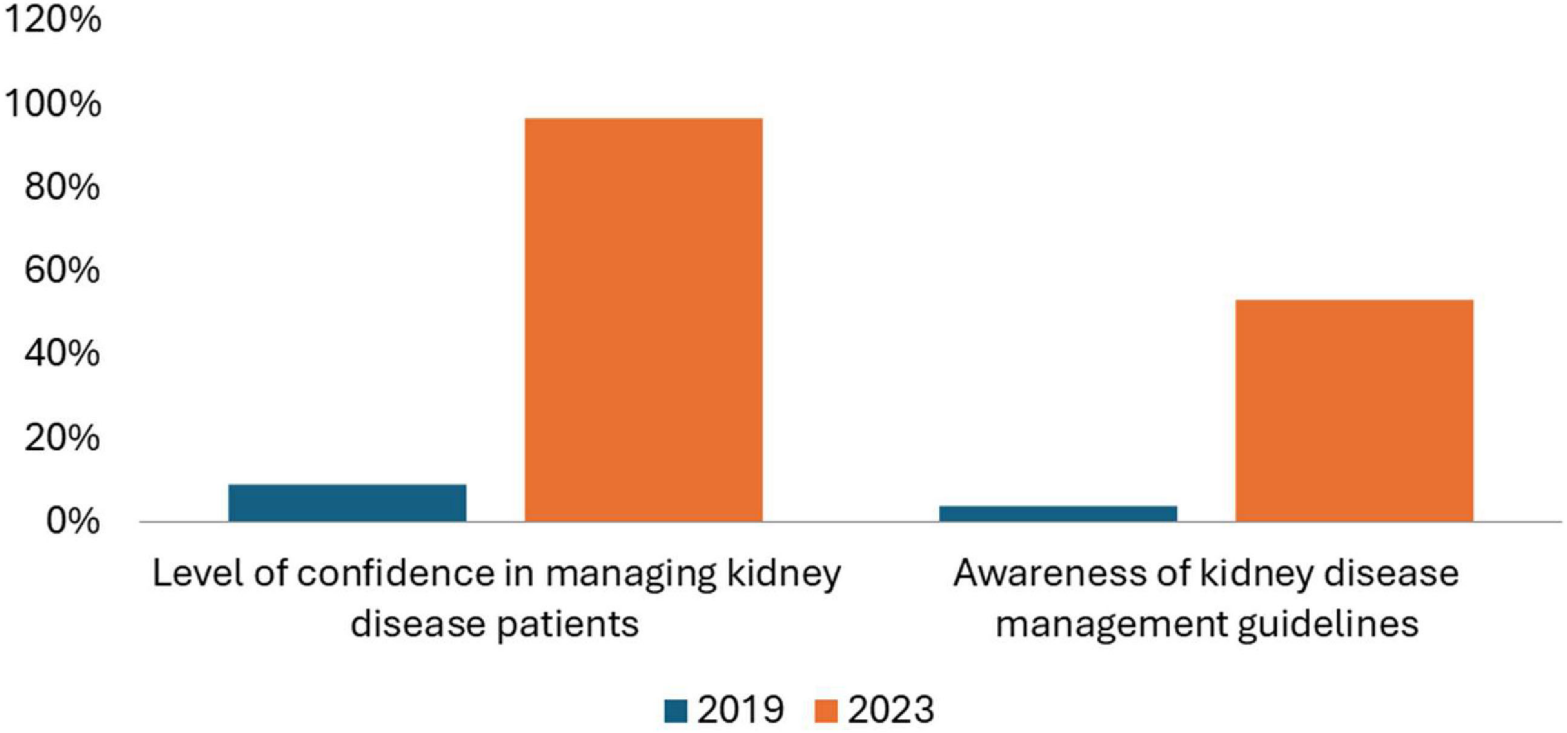
Comparison of level of confidence in managing kidney diseases and awareness of kidney disease management guidelines (2019 and 2023).

Among 30 internal medicine residents surveyed, 96.6% reported high confidence in managing kidney disease generally. Confidence in specific domains varied (Figure 2), and comparisons with the 2019 resident cohort showed significant improvements in confidence and guideline awareness (Figure 3). Overall, 36% of the study participants scored above 75% of the maximum score while 27% scored below 25%. However, in separate open-ended interviews, only 40 and 6.7% reported prior training in renal pathology and genetics, respectively. Access to nephrology journals was low (20%). Participation in educational sessions was high: 73.3% benefited from Zoom-based case conferences and 66.7% from journal clubs. Suggestions included starting a nephrology fellowship, covering topics like glomerular diseases and transplant care, and introducing a year-long curriculum.

### Brown fellow elective experience

**FIGURE 4:**
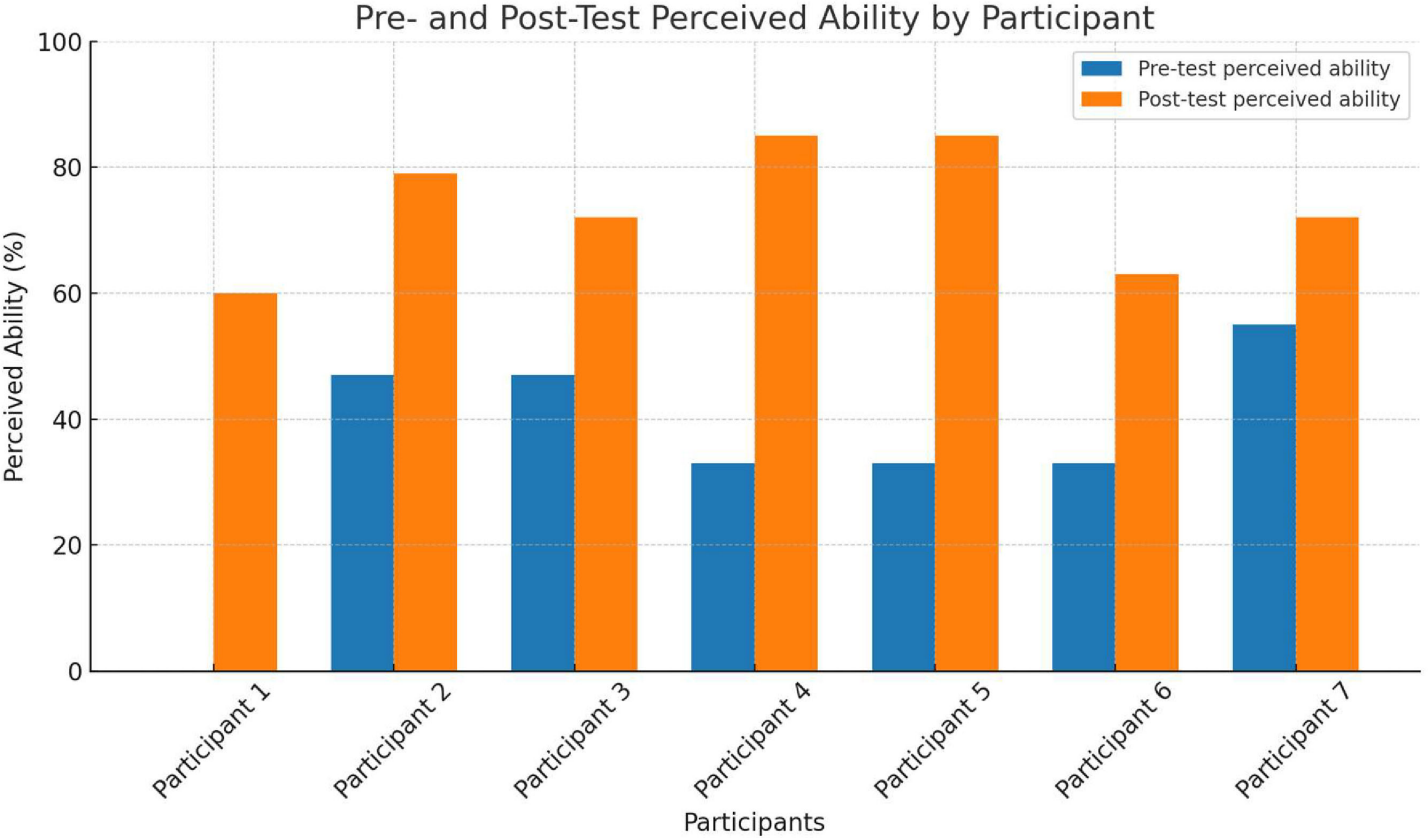
US nephrology fellow’s ability to contextualize key global health nephrology concepts pre- and post-exchange elective rotation.

Seven nephrology fellows completed 3-week electives in Eldoret between 2019 and 2023. Pre- and post-elective surveys showed an increase in perceived competency, from 36.1 to 62.0% on average. Figure 4 and Table 2 illustrate individual score changes.

**TABLE 2:** Impact of Kenya medical experience among trainees from U.S.

Identifier	Pre-trip score	Post-trip score
Participant 1	0.0 (0%)	15.0 (60%)
Participant 2	12.0 (48%)	20.0 (80%)
Participant 3	12.0 (48%)	18.0 (72%)
Participant 4	8.2 (33%)	21.5 (86%)
Participant 5	8.2 (33%)	16.0 (64%)
Participant 6	8.2 (33%)	20.0 (64%)
Participant 7	14.0 (56%)	18.0 (72%)
Mean	8.9 (36%)	18.3 (71%)

Values were expressed as the numerical number (maximum score = 25) and the % of maximum score.

## Discussion

This project illustrates how a sustained, multi-institutional partnership can help build nephrology capacity in a region with limited specialist resources. The collaboration between Moi University, Moi Teaching and Referral Hospital (MTRH), Brown University, and the Kenya Renal Association began in 2018 with a simple but ambitious goal: to strengthen nephrology education and training in Western Kenya. Over time, it has grown into a structured, dynamic program that blends local leadership with international academic support (Figure 1).

The early discussions were crucial. Stakeholders spent considerable time aligning priorities and identifying gaps. At the time, formal nephrology training opportunities were scarce, and most clinicians had limited exposure to current guidelines or case-based teaching. This reality shaped the initial focus on developing broad educational initiatives and continuing medical education for clinicians already in practice.

The first West Kenya Nephrology Conference in 2018 was a turning point. It brought together a diverse group of health professionals and quickly made clear the scale of the educational gap. Survey findings from the workshop showed low confidence in key clinical areas such as acute kidney injury (AKI) and hypertension management, as well as limited familiarity with standard nephrology guidelines. In response, several initiatives were launched in parallel. Moi University began hosting biweekly renal case conferences with support from North American faculty, and a formal nephrology curriculum was introduced. To accommodate the time difference between Kenya and the U.S., the conferences were scheduled for 3:30 PM local time, avoiding conflicts with other teaching sessions typically held at 8:00 AM. All Kenyan residents participated, but those who had rotated through the renal service were responsible for preparing cases for discussion, allowing both Kenyan and U.S.-based faculty to provide feedback and teaching. Annual workshops provided additional in-person training, while Brown University faculty contributed 2-week consultative services at MTRH that combined bedside teaching with collaborative patient care.

As the collaboration matured, virtual platforms played a growing role. Since October 2022, residents from Moi University have participated in Brown’s online case conferences and journal clubs. This has allowed continued educational engagement and helped create a sense of academic community that extends across continents.

The impact of these efforts is reflected in the longitudinal survey data. The 2019 baseline highlighted major educational needs, whereas the 2023 results showed marked increases in self-reported confidence, particularly in managing AKI and hypertension. While these are encouraging signs, gaps remained in areas like renal pathology and genetics, pointing to opportunities for further curriculum development and more advanced teaching modules. Importantly, the collaboration benefited both sides. Brown nephrology fellows reported gaining valuable global health experience, exposure to diverse clinical presentations, and improved cross-cultural communication skills thus an example of genuine bidirectional learning.

### Limitations

Notably, most of the 16 residents in the initial survey were second- and third-year trainees, whereas the 30 residents in the follow-up survey represented a more balanced distribution across all training levels. However, the higher proportion of first-year residents in the follow-up group would be expected to bias the results toward the null. Therefore, we believe our findings more convincingly demonstrate the positive impact of this collaborative training program. That said, the survey results may also be limited by the relatively small number of the resident class, and confounded by temporal factors, such as improved internet access, increased availability of web-based educational resources, and an enhanced nephrology curriculum for the 2023 cohort. Additionally, due to the absence of pre-existing validated instruments specific to our study topic, we developed a custom survey tool that has not undergone formal validation. Lastly, response bias—commonly encountered in surveys and interviews—should be considered when interpreting our results. To mitigate this, survey participation was voluntary and anonymous, and the questionnaires were intentionally kept short and concise.

Even with these limitations, the experience offers useful lessons. A structured, context-specific program, anchored in local leadership, supported by consistent faculty engagement, and sustained through both in-person and virtual interactions can help build specialty capacity in resource-limited settings. Looking ahead, strengthening content in pathology and genetics, incorporating objective knowledge assessments, and continuing to emphasize reciprocal exchange will be important next steps. Ultimately, this collaboration demonstrates how academic partnerships can move beyond one-time training to create lasting educational impact and shared growth.

### Future directions

Building on these successes, the collaboration aims to further enhance nephrology care in western Kenya. Priorities include community outreach and early CKD screening at the primary care level, supported by tele-nephrology and local capacity building. At the tertiary level, investments in renal pathology services, advanced imaging, and tissue typing laboratories are urgently needed, alongside efforts toward establishing a deceased donor kidney transplant program. Finally, the creation of a local nephrology fellowship program is a critical next step to strengthen the workforce and ensure sustainable expertise in the region.

## Data Availability

The raw data supporting the conclusions of this article will be made available by the authors, without undue reservation.
